# Metabolic Alteration of MCF-7 Cells upon Indirect Exposure to *E. coli* Secretome: A Model of Studying the Microbiota Effect on Human Breast Tissue

**DOI:** 10.3390/metabo13080938

**Published:** 2023-08-11

**Authors:** Reem H. AlMalki, Malak A. Jaber, Mysoon M. Al-Ansari, Khalid M. Sumaily, Monther Al-Alwan, Essa M. Sabi, Abeer K. Malkawi, Anas M. Abdel Rahman

**Affiliations:** 1 Department of Botany and Microbiology, College of Science, King Saud University, Riyadh 11451, Saudi Arabia; 439203044@student.ksu.edu.sa; 2 Pharmaceutical Medicinal Chemistry and Pharmacognosy, Faculty of Pharmacy and Medical Sciences, University of Petra, Amman 11196, Jordan; malak.jaber@uop.edu.jo; 3 Clinical Biochemistry Unit, Pathology Department, College of Medicine, King Saud University, Riyadh 11461, Saudi Arabia; ksumaily@ksu.edu.sa (K.M.S.); esabi@ksu.edu.sa (E.M.S.); 4 Cell Therapy and Immunobiology Department, King Faisal Specialist Hospital and Research Centre (KFSHRC), Riyadh 11211, Saudi Arabia; malwan@kfshrc.edu.sa; 5 Department of Chemistry and Biochemistry, Université Du Québec à Montréal, Montréal, QC H3C 3P8, Canada; malkawi.abeer@courrier.uqam.ca; 6 Metabolomics Section, Department of Clinical Genomics, Center for Genomics Medicine, King Faisal Specialist Hospital and Research Centre (KFSHRC), Riyadh 11211, Saudi Arabia

**Keywords:** microbiome, *E. coli* secretome, cancer, MCF-7 cells, metabolites, metabolomics, high-resolution mass spectrometry

## Abstract

According to studies, the microbiome may contribute to the emergence and spread of breast cancer. *E. coli* is one of the Enterobacteriaceae family recently found to be present as part of the breast tissue microbiota. In this study, we focused on the effect of *E. coli* secretome free of cells on MCF-7 metabolism. Liquid chromatography–mass spectrometry (LC-MS) metabolomics was used to study the *E. coli* secretome and its role in MCF-7 intra- and extracellular metabolites. A comparison was made between secretome-exposed cells and unexposed controls. Our analysis revealed significant alterations in 31 intracellular and 55 extracellular metabolites following secretome exposure. Several metabolic pathways, including lactate, aminoacyl-tRNA biosynthesis, purine metabolism, and energy metabolism, were found to be dysregulated upon *E. coli* secretome exposure. *E. coli* can alter the breast cancer cells’ metabolism through its secretome which disrupts key metabolic pathways of MCF-7 cells. These microbial metabolites from the secretome hold promise as biomarkers of drug resistance or innovative approaches for cancer treatment, either as standalone therapies or in combination with other medicines.

## 1. Introduction

Breast cancer (BC) is a complex disease influenced by various factors, including genetics, lifestyle, and environmental exposures [1]. Generally, the human body contains a diversity of bacteria, fungi, and viruses in/on the body, collectively known as the microbiota [2]. The breast microbiota refers to the collection of microorganisms that inhabit the breast tissue. The diversity of the breast microbiota has been studied extensively [3,4]. However, the link between the breast microbiome and breast cancer has become the main focus of researchers to identify specific microbial signatures for diseases among rising risk factors including the role of bacteria in the development and progression of the disease [5]. The presence of these microbial communities and their mechanisms may contribute to breast cancer, including inflammation and immune system dysregulation. Also, metabolites, including fatty acids, amino acids, and products of fermentation, appear to influence the breast tumor microenvironment (TME) and play a key role in the development and progression of the disease and thus could be potential targets for the treatment or prevention of breast cancer [6]. Recent studies have shown that the breast microbiota differs significantly in women with breast cancer compared to healthy women [7].

Interestingly, certain bacterial species have been found to be more prevalent in breast cancer tumors than in healthy breast tissue. It has been found that the presence of the bacterium *Fusobacterium nucleatum* in breast cancer tumors was associated with a more aggressive form of the disease and poor prognosis for patients [8,9].

We have shown recently that the *Escherichia coli* (*E. coli*) secretome linked to breast inflammation and cancer development affects significant metabolic processes, including fructose and mannose, sphingolipids, amino acids, fatty acids, amino sugar, nucleotide sugar, and pyrimidine [10]. 

Luminal A is a subtype of breast cancer that is known for its hormone receptor expression, making it the most prevalent form of breast cancer. In studies related to hormone-dependent signaling, the MCF-7 cell line is commonly used due to its estrogen receptor (ER) positivity [11]. The metabolism of MCF-7 cells can be divided into two main pathways. First, it has a high rate of basic cell metabolism, which involves the breakdown of carbohydrates, proteins, and lipids for energy production [12]. The second pathway is related to ER signaling and the synthesis of steroid hormones, particularly estradiol. This requires the catabolism of cholesterol and the synthesis of key steroid hormones such as progesterone and androgens [13]. MCF-7 cells can produce a range of proteins and other substances involved in signal transduction pathways, transcription, and cell growth, ultimately influencing tumorigenesis [14]. 

Metabolism supports various aspects of normal cell biology overall in our bodies. Changes to these fundamental features of cellular metabolism can result in multifactorial impacts at numerous levels, as in the initiation and progression of cancer [15]. Understanding cancer metabolism requires systematically applying analytical techniques to recognize and assess these metabolic anomalies. The growing technology known as metabolomics provides an overview of the metabolic network and its perturbations. It can thus be used to find new biomarkers for tracking therapy response and potential therapeutic targets [16,17]. In order to develop effective strategies for prevention, early detection, and treatment of breast cancer, it is crucial to fully comprehend the untargeted metabolomic changes that occur due to the crosstalk between the breast microbiota and cancer cells of luminal A subtype. A potential opportunity of research in this regard is exploring the impact of *E. coli* secretome on MCF-7 cells and its role in facilitating the interaction between the breast microbiota and cancer cells. By shedding light on this relationship, we can pave the way for novel approaches to tackle breast cancer.

## 2. Materials and Methods

### 2.1. Bacterial Supernatant Preparation

*Escherichia coli* (*E. coli*) from American Type Culture Collection (ATCC) 25922 was used. The secretome was prepared as follows. *E. coli* was grown in Luria–Bertani (LB) broth media for 24 h at 37 °C after collecting the supernatant and centrifugation at 10,000 rpm for 10 min. The supernatant was filtered using a disposable vacuum system with 0.22 µm pores. 

### 2.2. Cell Culture and Treatment

In this study, we obtained Michigan Cancer Foundation-7 (MCF-7) cells of luminal A subtype expressing estrogen receptor (ER) and progesterone receptor (PR) from ATCC (Manassas, VA, USA). These cells were routinely inspected for mycoplasma contamination using a PCR-based kit (Intron, Republic of Korea). Cells were cultured into T100 plates using DMEM/F-12 supplemented with 10% FBS, 1% penicillin/streptomycin, and 1% L-glutamine, then incubated at 37 °C with 5% CO_2_ in a humidified chamber. Once they reached confluence, MCF-7 cells were collected, after 4 passages at 70% of confluence, and counted using a light microscope (10 µL sample/10 µL dye), then counted using a hand tally counter (counting was conducted approximately with the same initial number of cells (1 × 10^6^ cells/mL). Treatment of cells was performed using 10% *E. coli* secretome in Serum-Free Media (SFM), while control cells were treated with 10% of pure LB media in SFM and incubated at different time points (0, 1, 2, 6, 8, and 24 h). Control was performed by having a parallel non-treated set of samples with the same number of experimental replicates to exclude any potential metabolomics profile associated with all the environmental and uncontrolled conditions including fluctuations in temperature, humidity, etc. Mainly using these measures, we excluded culture contaminants but not the direct effect of the *E. coli* supernatant. The other control was performed to identify profiling of 0 h treated vs. 0 h non-treated to exclude any media-related contamination and profile the *E. coli* secretome. This experiment was performed once, where each time point was represented in triplicate. In addition, 10% of media free of MCF-7 were cultured for 72 h to ensure the absence of contamination for quality control. 

### 2.3. Metabolomics Sample Preparation

To investigate the metabolic alterations occurring in our cell model, we followed a previously reported protocol to extract intra- and extracellular metabolites [10]. Briefly, in intracellular metabolism, medium was removed, and cells were washed with cold PBS followed by quenching in liquid nitrogen. The cells were extracted by 80% (*v*:*v*) MeOH:H_2_O and scraped using a cell scraper. The mixture was vortexed in a Thermomixer (Eppendorf, Germany) for 1 h at 600 rpm on 4 °C. The mixture was then spun down for 10 min at 4 °C, 10,000 rpm. The supernatants were transferred to new Eppendorf tubes. The extracellular metabolites were extracted by adding 50% (ACN: MeOH) to media and vortexed in a Thermomixer (Eppendorf, Germany) at 600 rpm at 4 °C for 1 h. The samples were spun down at 10,000 rpm, 4 °C for 10 min, and then the supernatant was transferred to new Eppendorf tubes. The intra- and extracellular extracts were evaporated completely in a Speed-Vac (Christ, Germany) and stored at −80 °C until LC-MS analysis.

### 2.4. LC-HRMS Metabolomics

The samples were reconstituted in 50% mobile phase A:B (A: 0.1% formic acid in dH_2_O, B: 0.1% formic acid in 50% MeOH and ACN) for untargeted metabolomics analyses using LCMS as previously reported [10]. Metabolites were acquired by a Waters Acquity ultra pressure liquid chromatography (UPLC) system coupled with a Xevo G2-S QTOF mass spectrometer equipped with an electrospray ionization source (ESI) on positive and negative (ESI+, ESI−). The metabolites were chromatographed using an ACQUITY UPLC XSelect (100 × 2.1 mm 2.5 μm) column (Waters Ltd., Elstree, UK). The mobile phases A and B were pumped to the column in a gradient mode (0–16 min 95–5% A, 16–19 min 5% A, 19–20 min 5–95% A, 20–22 min 95–95% A) at 300 μL/min flow rate. MS conditions were as follows: the source temperature was 150 °C, the desolvation temperature was 500 °C (ESI+) or 140 °C (ESI−), capillary voltages were 3.20 kV (ESI+) or 3 kV (ESI−), cone voltage was 40 V, desolvation gas flow was 800.0 L/h, and cone gas flow was 50 L/h. The collision energy of low and high functions was set off, at 10–50 V, respectively, in MS^E^ mode. The mass spectrometer was calibrated, as recommended by the vendor, with sodium formate in the range of 100–1200 Da in both ionization modes. Data Independent Acquisition (DIA) was collected in continuum mode with a Masslynx™ V4.1 workstation (Waters Inc., Milford, MA, USA). 

### 2.5. Data and Statistical Analyses

The MS raw data were processed following a standard pipeline starting from alignment based on the *m*/*z* value and the ion signals’ retention time, peak picking, and signal filtering based on the peak quality using the Progenesis QI v.3.0 software from Waters (Waters Technologies, Milford, MA, USA). Multivariate statistical analysis was performed using MetaboAnalyst v. 5.0 (McGill University, Montreal, QC, Canada) (http://www.metaboanalyst.ca, accessed on 5 June 2022) [18]. The imported datasets were normalized by median, Pareto-scaled, log-transformed to maintain their normal distribution, and then used to generate partial least squares-discriminant analysis (PLS-DA) and orthogonal partial least squares-discriminant analysis (OPLS-DA) models. The OPLS-DA models created were evaluated using the fitness of the model (R2Y) and predictive ability (Q2) values [19]. Univariate analysis was performed using Mass Profiler Professional software (Agilent, Santa Clara, CA, USA). One-way analysis of variance (ANOVA) with Tukey’s post-hoc and false discovery rate (FDR) *p* ≤ 0.05 was performed among time points. Volcano plot representation was used to identify significantly altered mass features based on a fold change (FC) cutoff of 2 and FDR *p* ≤ 0.05. Venn diagrams were developed using MPP Software (Agilent Inc., Santa Clara, CA, USA), and heatmap analysis for altered features was performed using the distance measure of Pearson; each experiment was performed in triplicate at 24 h, outliers were minimal, and already tested. 

### 2.6. Metabolites Identification

The significant features obtained from intra- and extracellular metabolites were annotated using the Human Metabolome Database (HMDB) based on the accurate precursor mass, the fragmentation pattern, and isotopic distribution [20]. The *E. coli* database was used to identify *E. coli* metabolites in the extracellular media samples [21]. Exogenous compounds, such as drugs and food additives, were eliminated manually from the final list.

## 3. Results

### 3.1. Metabolites of E. coli Secretome

**Figure 1:**
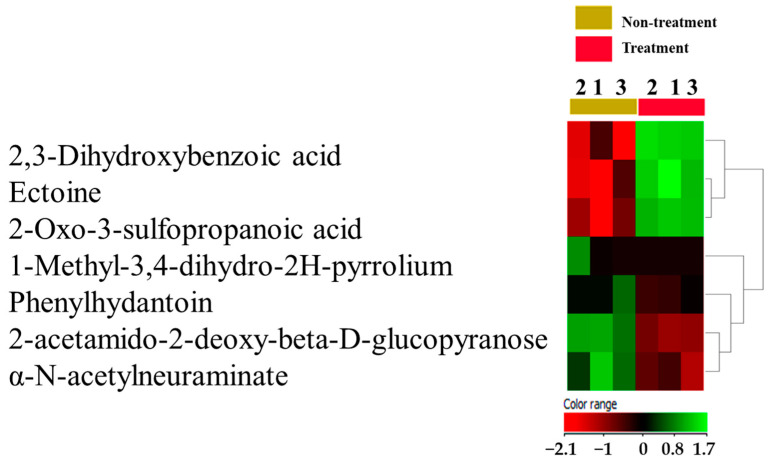
*E. coli*-related secretome identification upon treatment. A heatmap and hierarchical cluster analysis showed only 7 *E. coli*-related excreted metabolites in conditioned media of MCF-7 cells compared to the control.

Initially, *E. coli* growth density was measured (OD = 1.5 in 1 mL/LB) to prepare the *E. coli* secretome in a reproducible fashion. The *E. coli*-secreted metabolites in conditioned media were profiled by comparing their expression in treated and non-treated samples at baseline exposure (moderated *t*-test, cut-off: no correction *p*-value ≤ 0.05, and FC of 2). A total of 672 metabolites were revealed at baseline, where 185 and 487 were up- and down-regulated, respectively. The *E. coli* metabolome database identified 26 metabolites. Only seven out of 26 metabolites were identified and secreted from *E. coli* (Figure 1). These metabolites potentially affect the MCF-7 metabolism as represented in the heatmap developed on *E. coli*-related metabolites based on Pearson’s correlation coefficient and average linkage methods. The data are summarized in Table S1. 

### 3.2. Mass Ion Detection and Dysregulated Intracellular Metabolites after Treating MCF-7 Cells with E. coli Secretome at Different Time Points

**Figure 2:**
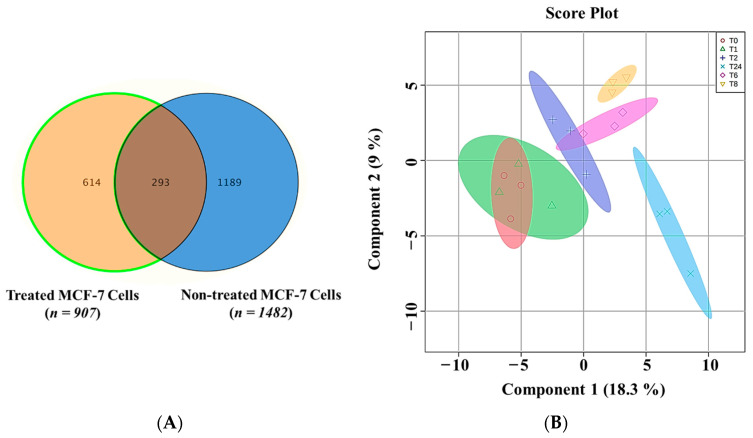
(**A**) A Venn diagram represents the relation between significantly dysregulated intracellular ions in treated MCF-7 with *E. coli* secretome (*n* = 907) and non-treated cells (*n* = 1482) at different time points (0, 1, 2, 6, 8, and 24 h). (**B**) Sample clustering and group separation. PLS-DA of 614 features of MCF-7 cells that were treated with *E. coli* secretome at different time points (0, 1, 2, 6, 8, and 24 h).

In intracellular extracts, 12,657 mass ions were obtained in positive and negative ionization modes. Of these ions, 7833 and 4824 were detected in positive and negative ionization modes, respectively. The data were deposited in Metabolomics workbench (ST002715). One-way ANOVA (Tukey’s post-hoc, FDR *p* ≤ 0.05) was carried out in treated and untreated cells at various time points after missing values were excluded and imputed. A total of 907 and 1482 ions were significantly dysregulated in treated and non-treated cells at various time points, respectively. The Venn diagram in Figure 2A represents the dysregulated ions after excluding culture media and incubation background-related metabolites. A total of 293 ions were affected by treatment and culture conditions.

The remaining 614 ions were significantly dysregulated in response to the *E-coli* secretome exposure. These ions (*n* = 614) were used to represent the changes at various time points. Figure 2B represents the PLS-DA plot, showing study samples clustering and group separation at different treatment time points. 

**Figure 3:**
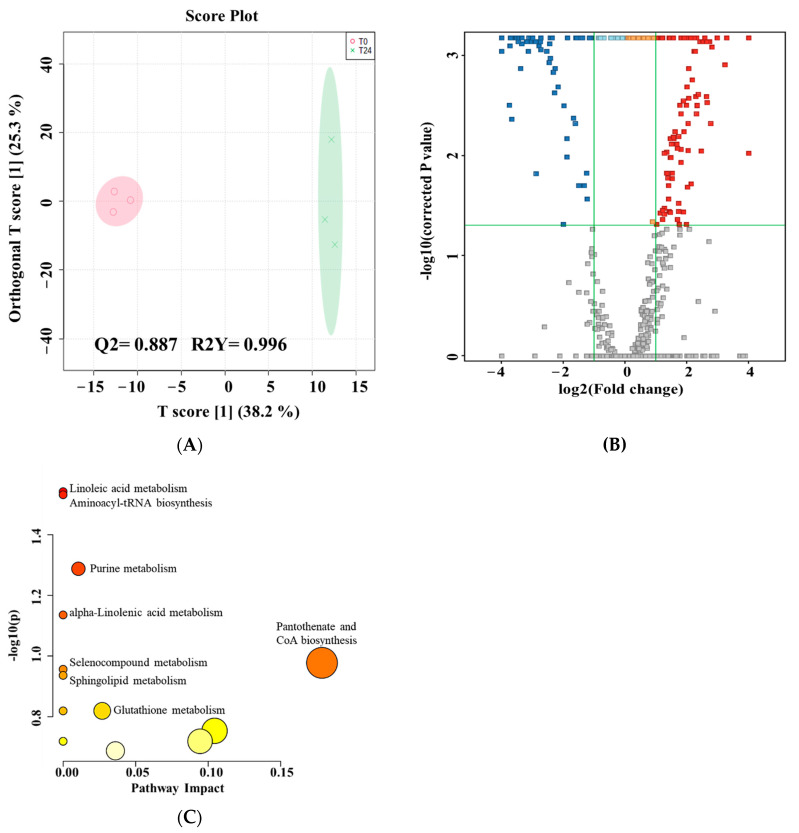
Dysregulated intracellular metabolites between MCF-7 cells pre- and 24 h post-treatment with *E. coli* secretome. (**A**): An OPLS-DA model of MCF-7 cells treated with *E. coli* secretome shows a clear separation between pre- and 24 h post-treatment. The robustness of the created model was evaluated by the fitness of the model (R2Y = 0.996), and predictive ability (Q2 = 0.887) values in a larger dataset (*n* = 1000). (**B**): Univariate analysis using Volcano plot based on culture background-free features (*n* = 614) showed 93 (red), and 67 (blue) metabolites were up- and down-regulated 24 h post-treatment compared to pre-treatment, respectively (cut-off: FDR *p* ≤ 0.05, and FC 2). (**C**): Pathway analysis of significant metabolites dysregulated in treated MCF-7 cells with *E. coli* secretome after 24 h.

As demonstrated in the multivariant analysis, the greatest amount of metabolic alteration was after 24 h of treatment. A binary comparison after 24 h of treatment with the corresponding control was performed using multi- and univariate analyses; the OPLS-DA model (Figure 3A) showed the group separation and sample clustering between the 0 and 24 h of treatment. The model produced goodness of prediction (Q2: 0.887) and the fitness of the model (R2Y: 0.996). Univariate analysis using Volcano plot (cut-off: FDR *p* ≤ 0.05, and FC 2) revealed 160 ions as significantly dysregulated. Of these 160 ions, 93 ions were up-regulated, and 67 were down-regulated after 24 h post-treatment compared to the corresponding control sample (Figure 3B). Only 79 metabolites were identified from the 160 significant ions following annotation with the HMDB. After manually excluding the exogenous metabolites, the remaining 31 endogenous metabolites were used for further pathway analysis. A total of 20 and 10 metabolites were up- and down-regulated after 24 h of treatment. These metabolites are summarized in Table 1. Perturbation in these metabolites resulted in changes in 13 metabolic pathways (Figure 3C). Pantothenate and CoA biosynthesis was the most affected pathway based on pathway impact.

### 3.3. Mass Ion Detection and Dysregulated Extracellular Metabolites after Treating MCF-7 Cells with E. coli Secretome

The metabolomics profile of the extracellular extracts detected 12,997 mass ions in positive and negative ionization modes. After missing values exclusion, one-way ANOVA (Tukey’s post-hoc FDR *p* ≤ 0.05) was used to detect the significantly changed ions between different time points in MCF-7 treated and non-treated cells. The analysis revealed that 1948 and 3128 ions were significantly dysregulated at different time points in treated and non-treated groups, respectively. The culture and incubation background-associated ions were eliminated from the treated samples using a Venn diagram. Of the dysregulated ones, 821 ions detected in treated cells were retained for further analyses, as displayed in Figure S1A.

As mentioned earlier, most metabolic changes happened 24 h post-treatment. Thus, the binary comparison between pre-and 24 h post-treatment using Volcano plot (Cut-off: FDR *p* ≤ 0.05, and FC 2) reveals that 437 metabolites were significantly dysregulated, of which 159 and 278 metabolites were up-and down-regulated 24 h post-treatment compared to control, respectively (Figure S1B,C). Only 55 out of 243 metabolites were identified as endogenous metabolites (Table 2). Pathway analysis revealed that the most affected pathways include one carbon pool by folate, nicotinate and nicotinamide metabolism, pyruvate metabolism, ether lipid metabolism, folate biosynthesis, pentose, and glucuronate interconversions, cysteine and methionine metabolism, and tryptophan metabolism (Figure S1D).

## 4. Discussion

The scientific community has acknowledged that the breast can provide a favorable environment for the growth of bacteria since it is composed primarily of fatty tissue and has significant lymphatic drainage, lobules, and vasculature [8,22]. However, the microbiome distribution varies considerably from healthy subjects to cancer patients [22,23,24]. Patients with BC exhibited greater relative phyla abundances, including *staphylococcus*, *bacillus*, *Enterobacteriaceae*, *firmicutes*, *actinobacteria*, *bacteroidetes* and *proteobacteria*, and others [8,25,26,27]. Since a higher abundance of *E. coli* was detected in women with BC than in healthy controls, this study employed metabolomics to reveal the impact of *E. coli* secretome on the MCF-7 cellular biological processes.

The microbiome can modify the physiology of the host cells through metabolites that enter the circulation and reach their target cells, similar to human hormones [24]. These microbial metabolites could regulate the TME; hence, a deeper comprehension of how microbial pathogens affect BC can improve future prevention, diagnosis, and treatment options.

### 4.1. Metabolites Related to E. coli Secretome

The current study found seven metabolites related to *E. coli* secretome that might play important roles in BC pathogenesis or protection. Our findings showed that 2,3-dihydroxybenzoic acid (DHBA) was elevated in the *E. coli* secretome. DHBA is a siderophore secreted by *E. coli* and other pathogens under conditions of low iron availability to increase their virulence. DHBA sequesters iron from the host, which is known to be a severely iron-restricted growth environment, and provides this essential metal nutrient to microbes [28,29].

An interesting finding in our study is the elevated α-N-acetylneuraminate found in the *E. coli* secretome, also known as sialic acids. *E. coli* can grow on Neu5Ac as a carbon source [30]. *E. coli* can use these sialic acids to capsule their polysaccharides to mimic the host cells leading to a dampening of immune responses and so increasing their survival [31,32]. This could be one of many reasons why higher percentages of *E. coli* colonies were detected in BC tissue compared to healthy tissue.

Additionally, ectoine in the *E. coli* secretome was found to be elevated; this is a natural metabolite that has a role as an osmolyte, which helps *E. coli* avoid deleterious increases in ion concentration and maintain cytoplasmic electroneutrality [33]. Moreover, ectoine has induced apoptosis in lung cancer cells without toxic effects on normal cells [34]. Thus, ectoine could have a potential role in protecting from cancer.

Moreover, our analysis showed that 2-oxo-3-sulfopropanoic acid was up-regulated in *E. coli* secretome. 2-oxo-3-sulfopropanoic acid plays a key role in amino acid metabolism [35]. As a result, the metabolites of *E. coli* may influence the TME and contribute to the development or prevention of cancer. In turn, TME may influence the virulence of these pathogens.

### 4.2. Dysregulated Intracellular Metabolites after Treating MCF-7 Cells with E. coli Secretome

Acetylated methionine and lysine were up-regulated in MCF-7 treated cells, which are crucial for proper mitochondrial protein acetylation. The degree of acetylation is influenced by nutrient availability and cellular metabolic status. Increased acetylation is associated with physiological conditions that result in higher levels of acetyl-CoA, such as fasting, calorie restriction, a high-fat diet, and ethanol intoxication, to avoid mitochondria overfeeding [36]. Acetyl coenzyme A carboxylase alpha (ACCA) is a biotin-dependent enzyme that catalyzes the carboxylation of acetyl-CoA to produce malonyl-CoA, which is used in fatty acid synthase. As a protective mechanism, the tumor suppressor gene breast cancer susceptibility gene 1, also known as BRCA1, interacts with ACCA and stabilizes the inactive state, which prevents tumor cell anabolism, suppresses the malignant phenotype, and raises Acetyl-CoA levels [37]. In addition, a low level of intracellular methionine was detected. Methionine is consumed in the methionine cycle to generate S-adenosylmethionine (SAM), which serves as a methyl donor; increased methionine cycle results in an overabundance of SAM that can lead to enhanced tumor growth [38].

According to a study, women with very low AST/ALT ratios had higher BC risks than women with moderate AST/ALT ratios resulting in the detection of a higher level of alanine [39]. This increased synthesis of alanine may be intended to counteract the anti-carcinogenic effect caused by some of the *E. coli* secretomes discussed previously. However, it has been demonstrated that the alanine content of tumor tissues correlates favorably with tumor malignancy, and thus *E. coli* secretome may promote the growth and virulence of tumors. This might increase the sensitivity of cancer cells to ALT inhibitors. Inhibition of ALT effectively slows cancer growth by counteracting the Warburg effect. When the Warburg effect is reduced in cancer cells, compensatory activation of mitochondrial metabolism by activating AMP-activated protein kinase is initiated. This will increase the respiration rates and mitochondrial production of reactive oxygen species, negatively affecting cancer growth [40].

In addition, we showed several lipids such as induction of diglyceride, phosphatidylcholine, and phosphatidylinositol keep up with the increased demand during progression in response to *E. coli* secretome. Lipids’ high production, storage, and absorption contribute to cancer progression. The changing of lipid metabolism in cancer has been connected to the activation of oncogenic signaling pathways and cross-communication with the tumor microenvironment [41].

### 4.3. Dysregulated Extracellular Metabolites after Treating MCF-7 Cells with E. coli Secretome

Significant amounts of lactic acid produced during aerobic glycolysis and glutaminolysis are released into the TME. Cancer cells’ overexcretion of lactic acid prevents intracellular acidification and upregulates the glycolytic pathway [42]. Moreover, it has been suggested that lactate functions as an oncometabolite in the MCF7 human BC cell line because it boosts the transcriptional activity of MYC, a potent facilitator of carcinogenesis associated with poor prognosis in malignancies [43].

In addition, our data showed increase in β-oxidation and fatty acid catabolism documented in tumor cells, which is an adaptive response to nutrient deprivation and environmental stressors [44]. The acidity of the TME causes a metabolic adaptation of the tumor cell population and promotes β-oxidation as a metabolic strategy (the Corbet−Feron Effect) [45]. Also, the increased acylcarnitines level suggests incomplete fatty acid oxidation due to increased β-oxidation, which exceeds the tricarboxylic acid cycle’s capacity. However, decreased lactic acid concentration in the extracellular extract of treated cells could be due to lactate metabolism by the *E. coli* secretome. Therefore, these perturbations in key regulatory processes could lead to inhibited tumor growth. 

N-lactoyl-tryptophan and indole-3-carboxaldehyde were downregulated, which is recognized as a disturbance in tryptophan metabolism after the MCF-7 cells’ treatment with *E. coli* secretome. An elevated level of N-lactoyl-tryptophan was associated with mitochondrial dysfunction in mitochondrial encephalomyopathy lactic acidosis [46]. The decrease in lactic acid concentration discussed above is more likely to cause a drop in N-lactoyl-amino acids synthesis.

Importantly, tumor cells have been found to contain significant levels of purine metabolites [47]. The complementary salvage method and the de novo biosynthetic pathway are used in mammalian cells to create purine nucleotides. Most of the cellular needs for purine are often met by the complementary salvage route, which recycles the bases that have been broken down. In the circumstances with a larger requirement for purine nucleotides, such as proliferating cells and tumor cells, the de novo manufacturing process is crucial to replenish the purine pool [48]. Upon the treatment with *E. coli* secretome, two purine metabolites were dysregulated in MCF 7 extracellular media, namely SAICAR and inosine, which are metabolites of the de novo purine biosynthetic pathway and its salvage process, respectively. This suggests that the microbiological secretome impact on cancer cells might be attributed to tampering with the nucleotide metabolism.

## 5. Conclusions

Tumor cells can survive in the face of unfavorable environmental conditions due to cancer’s metabolic flexibility. A metabolite’s ability to change in response to internal or external perturbations is a requirement for metabolic plasticity. Although the *E. coli* secretome could have some beneficial effects against cancer growth and propagation, the MCF-7 cell’s responses are able to reverse these alterations by lipid metabolic reprogramming, increased ALT activity, and acylation. This may in turn render the cancerous cells more aggressive. However, due to the metabolic network’s complicated structure, locating regulatory nodes within it is attractive additional research that could help us to understand the overall picture fully.

## Figures and Tables

**Table 1:** Intracellular endogenous metabolites which were identified as significantly dysregulated. Binary comparison of MCF-7 with *E. coli* secretome in 24 h post-treatment compared to pre-treatment. FDR *p*-value < 0.05, FC cutoff is 2.

Compound	HMDB ID	Compound Name	RT	*m*/*z*	*p* Value	FC	Log FC	Regulation
0.65_518.9934 *m*/*z*	HMDB0041706	Caffeic acid 3-O-sulfate	0.65	518.99	0.00	2.96	1.57	up
0.67_259.0768 *m*/*z*	HMDB0032552	Vanillin 1,2-butylene glycol acetal	0.67	259.08	0.00	16.00	4.00	up
0.83_341.1082 *m*/*z*	HMDB0041306	Methyl 2-(methylthio)butyrate	0.83	341.11	0.00	4.38	2.13	up
0.89_487.2109 *m*/*z*	HMDB0296919	DG(2:0/PGJ2/0:0)	0.89	487.21	0.00	2.33	1.22	up
1.78_134.0454 *m*/*z*	HMDB0000161	L-Alanine	1.78	134.05	0.00	2.33	1.22	up
12.39_409.1679 *m*/*z*	HMDB0031920	9-Hydroxycalabaxanthone	12.39	409.17	0.01	3.65	−1.87	down
14.60_272.1178 n	HMDB0035191	(2S,4R)-4-(9H-Pyrido[3,4-b]indol-1-yl)-1,2,4-butanetriol	14.60	589.23	0.02	4.40	2.14	up
3.12_453.0646 *m*/*z*	HMDB0001508	dADP	3.12	453.06	0.01	2.77	1.47	up
3.34_366.2033 *m*/*z*	HMDB0294083	CDP-DG(PGF1alpha/i-19:0)	3.34	366.20	0.01	2.55	1.35	up
4.35_115.0542 *m*/*z*	HMDB0002222	3-Methylphenylacetic acid	4.35	115.05	0.01	2.76	1.46	up
4.92_171.1124 *m*/*z*	HMDB0000446	N-alpha-Acetyl-L-lysine	4.92	171.11	0.01	2.35	−1.23	down
5.31_733.6413 *m*/*z*	HMDB0298285	DG(18:3(9,11,15)-OH(13)/0:0/a-25:0)	5.31	733.64	0.04	2.21	1.14	up
5.33_738.4847 *m*/*z*	HMDB0008240	PC(18:4(6Z,9Z,12Z,15Z)/18:4(6Z,9Z,12Z,15Z))	5.33	738.48	0.04	2.69	1.43	up
5.35_731.6062 *m*/*z*	HMDB0001348	SM(d18:1/18:0)	5.35	731.61	0.00	2.24	1.16	up
5.59_729.0708 *m*/*z*	HMDB0000934	Uridine diphosphate acetylgalactosamine 4-sulfate	5.59	729.07	0.03	3.35	1.74	up
5.96_645.3717 *m*/*z*	HMDB0298359	DG(PGE2/i-12:0/0:0)	5.96	645.37	0.00	3.61	−1.85	down
6.18_187.0280 *m*/*z*	HMDB0304115	3-butenylglucosinolate	6.18	187.03	0.00	16.82	−4.07	down
6.59_492.2480 *m*/*z*	HMDB0278346	PI(PGJ2/22:6(4Z,7Z,10Z,13Z,16Z,19Z))	6.59	492.25	0.01	3.01	1.59	up
7.68_926.4949 *m*/*z*	HMDB0276269	PI(PGJ2/16:2(9Z,12Z))	7.68	926.49	0.01	2.95	1.56	up
7.69_108.0273 *m*/*z*	HMDB0028768	Cysteinyl-Alanine	7.69	108.03	0.00	6.27	2.65	up
7.69_174.0575 *m*/*z*	HMDB0011745	N-Acetyl-L-methionine	7.69	174.06	0.00	6.48	2.70	up
7.69_263.1203 *m*/*z*	HMDB0040672	3-Oxo-alpha-ionol 9-[apiosyl-(1->6)-glucoside]	7.69	263.12	0.00	5.74	2.52	up
8.00_160.0420 *m*/*z*	HMDB0001015	N-Formyl-L-methionine	8.00	160.04	0.00	3.97	1.99	up
8.00_205.9999 *m*/*z*	HMDB0001000	dUDP	8.00	206.00	0.01	3.34	1.74	up
8.00_347.0763 *m*/*z*	HMDB0011691	Cytidine 2′,3′-cyclic phosphate	8.00	347.08	0.00	4.14	2.05	up
8.41_403.0972 *m*/*z*	HMDB0001117	4′-Phosphopantothenoylcysteine	8.41	403.10	0.00	5.35	−2.42	down
8.94_365.1163 *m*/*z*	HMDB0040760	4,4′-Dihydroxy-5,5′-diisopropyl-2,2′-dimethyl-3,6-biphenyldione	8.94	365.12	0.00	16.00	−4.00	down
9.87_158.0278 *m*/*z*	HMDB0029508	Laccaic acid D	9.87	158.03	0.00	15.79	−3.98	down
9.89_107.5117 *m*/*z*	HMDB0000682	Indoxyl sulfate	9.89	107.51	0.00	16.00	−4.00	down
9.89_114.0363 *m*/*z*	HMDB0000696	L-Methionine	9.89	114.04	0.00	7.97	−2.99	down
9.89_178.0233 *m*/*z*	HMDB0006555	dIMP	9.89	178.02	0.00	21.14	−4.40	down

**Table 2:** Dysregulated extracellular endogenous metabolites that were secreted in culture media after treating MCF-7 cells with *E. coli* secretome. Binary comparison of MCF-7 cells treated with *E. coli* secretome in 24 h post-treatment compared to pre-treatment. FDR *p*-value < 0.05, FC cutoff is 2.

Compound	HMDB ID	Compound Name	RT	*m*/*z*	*p* Value	FC	Log FC	Regulation
3.53_340.1895 *m*/*z*	HMDB0060988	5-hydroxypropafenone	3.53	340.19	0.00	2.05	1.03	up
3.53_461.1963 *m*/*z*	HMDB0260498	MG(20:5(7Z,9Z,11E,13E,17Z)-3OH(5,6,15)/0:0/0:0)	3.53	461.20	0.00	2.02	1.01	up
3.75_229.1521 *m*/*z*	HMDB0011174	Isoleucylproline	3.75	229.15	0.00	2.68	1.42	up
4.09_438.2217 *m*/*z*	HMDB0240776	O-(13-Carboxytridecanoyl)carnitine	4.09	438.22	0.00	2.14	1.10	up
4.49_591.2626 *m*/*z*	HMDB0029005	Phenylalanylthreonine	4.49	591.26	0.00	2.06	1.05	up
5.49_429.2150 *m*/*z*	HMDB0011154	LysoPA(P-16:0/0:0)	5.49	429.22	0.00	2.00	−1.00	down
5.58_311.0837 *m*/*z*	HMDB0062178	N-lactoyl-Tryptophan	5.58	311.08	0.00	4.31	−2.11	down
5.94_197.0562 *m*/*z*	HMDB0004194	N1-Methyl-4-pyridone-3-carboxamide	5.94	197.06	0.00	2.31	1.21	up
6.02_307.1754 n	HMDB0241867	4-Phenylbutanoylcarnitine	6.02	352.17	0.00	2.24	1.16	up
6.17_514.2649 *m*/*z*	HMDB0011475	LysoPE(0:0/18:1(11Z))	6.17	514.26	0.00	2.24	1.16	up
6.53_392.2754 n	HMDB0039019	3-Hydroxy-10′-apo-b,y-carotenal	6.53	437.27	0.00	2.14	−1.10	down
6.98_566.2758 *m*/*z*	HMDB0240604	LysoPS(18:2(9Z,12Z)/0:0)	6.98	566.28	0.00	2.04	1.03	up
7.06_179.0549 *m*/*z*	HMDB0000190	L-Lactic acid	7.06	179.05	0.00	16.00	−4.00	down
7.08_315.1322 *m*/*z*	HMDB0242134	3-Aminopiperidine-2,6-dione	7.08	315.13	0.00	8.98	−3.17	down
7.09_884.4338 *m*/*z*	HMDB0281253	PS(TXB2/16:1(9Z))	7.09	884.43	0.00	2.32	1.21	up
7.15_961.2977 *m*/*z*	HMDB0060299	(1R)-Glutathionyl-(2R)-hydroxy-1,2-dihydronaphthalene	7.15	961.30	0.00	15.28	3.93	up
7.21_528.2782 *m*/*z*	HMDB0241876	(5Z)-7-[(1R,2R,5S)-5-Hydroxy-2-[(1E,3S,5Z)-3-hydroxyocta-1,5-dien-1-yl]-3-oxocyclopentyl]hept-5-enoylcarnitine	7.21	528.28	0.00	2.28	−1.19	down
7.25_1071.2554 *m*/*z*	HMDB0060783	6-beta-Hydroxy-mometasone furoate	7.25	1071.26	0.00	2.95	1.56	up
7.25_730.1474 *m*/*z*	HMDB0031996	Licorice glycoside E	7.25	730.15	0.01	3.04	1.60	up
7.27_307.1102 *m*/*z*	HMDB0032673	15-Octadecene-9,11,13-triynoic acid	7.27	307.11	0.00	6.31	2.66	up
7.27_334.1092 *m*/*z*	HMDB0241039	2,3-dimethylidenepentanedioylcarnitine	7.27	334.11	0.00	4.30	2.10	up
7.28_375.0968 *m*/*z*	HMDB0001272	Nicotine glucuronide	7.28	375.10	0.00	3.09	1.63	up
7.33_285.0968 *m*/*z*	HMDB0061112	3-Carboxy-4-methyl-5-propyl-2-furanpropionic acid	7.33	285.10	0.00	3.77	1.91	up
7.70_108.0272 *m*/*z*	HMDB0028768	Cysteinyl-Alanine	7.70	108.03	0.00	4.76	2.25	up
7.70_146.0620 *m*/*z*	HMDB0012267	N-Succinyl-L,L-2,6-diaminopimelate	7.70	146.06	0.00	4.14	2.05	up
7.70_263.1212 *m*/*z*	HMDB0001129	N-Acetylmannosamine	7.70	263.12	0.00	6.10	2.61	up
7.97_135.0497 *m*/*z*	HMDB0060810	cyclic 6-Hydroxymelatonin	7.97	135.05	0.00	2.22	1.15	up
7.97_969.9324 *m*/*z*	HMDB0043342	TG(15:0/22:1(13Z)/24:0)	7.97	969.93	0.01	2.92	1.55	up
7.99_185.0708 *m*/*z*	HMDB0000472	5-Hydroxy-L-tryptophan	7.99	185.07	0.00	2.81	1.49	up
8.00_206.0042 *m*/*z*	HMDB0001000	dUDP	8.00	206.00	0.00	2.27	1.19	up
8.61_1019.0126 *m*/*z*	HMDB0061723	Carbovir Triphosphate	8.61	1019.01	0.01	2.06	−1.04	down
8.89_169.0965 *m*/*z*	HMDB0060427	Acetone cyanohydrin	8.89	169.10	0.00	11.35	−3.50	down
8.89_255.1118 *m*/*z*	HMDB0304210	5,6-dihydrothymine	8.89	255.11	0.00	9.76	−3.29	down
8.89_391.1105 *m*/*z*	HMDB0004308	7,9-Dimethyluric acid	8.89	391.11	0.00	16.95	−4.08	down
8.89_478.1285 *m*/*z*	HMDB0001056	Dihydrofolic acid	8.89	478.13	0.00	16.00	−4.00	down
8.89_579.0252 *m*/*z*	HMDB0304422	N-acetylglutamyl-phosphate	8.89	579.03	0.00	7.39	−2.89	down
8.99_289.0995 *m*/*z*	HMDB0029737	Indole-3-carboxaldehyde	8.99	289.10	0.00	22.77	−4.51	down
8.99_994.1592 *m*/*z*	HMDB0300998	Undeca-3,5,7-trienedioyl-CoA	8.99	994.16	0.00	16.00	−4.00	down
9.11_288.1432 *m*/*z*	HMDB0240764	2-Ethylacryloylcarnitine	9.11	288.14	0.00	16.00	−4.00	down
9.16_865.5139 *m*/*z*	HMDB0268808	PG(5-iso PGF2VI/18:0)	9.16	865.51	0.00	2.29	1.20	up
9.17_1195.6690 *m*/*z*	HMDB0002596	Deoxycholic acid 3-glucuronide	9.17	1195.67	0.00	2.29	1.20	up
9.69_207.0898 n	HMDB0000512	N-Acetyl-L-phenylalanine	9.69	208.10	0.00	2.55	−1.35	down
9.87_1079.6930 *m*/*z*	HMDB0117448	CL(8:0/10:0/10:0/i-19:0)	9.87	1079.69	0.00	5.64	−2.50	down
9.87_114.0362 *m*/*z*	HMDB0000696	L-Methionine	9.87	114.04	0.00	9.10	−3.19	down
9.87_160.0422 *m*/*z*	HMDB0001015	N-Formyl-L-methionine	9.87	160.04	0.00	8.49	−3.09	down
9.87_182.0247 *m*/*z*	HMDB0006409	Tyramine-O-sulfate	9.87	182.02	0.00	10.10	−3.34	down
9.87_198.0525 *m*/*z*	HMDB0059660	sn-glycero-3-Phosphoethanolamine	9.87	198.05	0.00	9.64	−3.27	down
9.87_354.2685 *m*/*z*	HMDB0295990	DG(22:5(4Z,7Z,10Z,13Z,19Z)-O(16,17)/0:0/18:0)	9.87	354.27	0.00	16.00	−4.00	down
9.87_433.5980 *m*/*z*	HMDB0300835	4-Methylpentanoyl-CoA	9.87	433.60	0.00	9.67	−3.27	down
9.87_578.0937 *m*/*z*	HMDB0012278	Phosphoribulosylformimino-AICAR-P	9.87	578.09	0.00	19.62	−4.29	down
9.91_213.0398 n	HMDB0032357	N-Lactoyl ethanolamine phosphate	9.91	178.03	0.00	10.06	−3.33	down
9.91_260.0232 *m*/*z*	HMDB0011725	5-Sulfosalicylic acid	9.91	260.02	0.00	12.30	−3.62	down
9.91_419.0582 *m*/*z*	HMDB0000797	SAICAR	9.91	419.06	0.00	5.82	−2.54	down
9.91_512.3994 *m*/*z*	HMDB0011187	TG(8:0/8:0/8:0)	9.91	512.40	0.00	16.00	−4.00	down
9.91_513.0681 *m*/*z*	HMDB0006354	Deoxythymidine diphosphate-L-rhamnose	9.91	513.07	0.00	16.00	−4.00	down

## Data Availability

The raw data of this study were deposited at Metabolomics Workbench on 22 June 2023 and can be accessed under accession number ST002715.

## Supplementary Materials

The following supporting information can be downloaded at: https://www.mdpi.com/article/10.3390/metabo13080938/s1, Figure S1: Dysregulated extracellular metabolites between MCF-7 cells pre-and 24 h post-treatment with *E. coli* secretome. (**A**) A Venn diagram represents the relation between significantly dysregulated ex-tracellular ions in treated MCF-7 with *E. coli* secretome (n = 1948) and non-treated cells (n = 3128) at different time points (0, 1, 2, 6, 8, and 24 h), (**B**) An OPLS-DA model represents the separation between pre- and 24 h post-treatment samples based on selected 821 extracellular ions. The ro-bustness of the created model was evaluated by the fitness of the model (R2Y = 0.999) and predic-tive ability (Q2 = 0.984) values in a larger dataset (n = 1000). (**C**) Volcano plot revealed 437 sig-nificantly dysregulated metabolites, where 159 (in red) and 278 (in blue) ions were up- and down-regulated in 24 h post-treatment compared to control, respectively (Cut-off: FDR ≤ 0.05, and FC 2). (**D**) Pathway analysis for the significant metabolites dysregulated that secreted in cul-ture media after treating MCF-7 cells with *E. coli* secretome. 56 metabolites were ultimately iden-tified as endogenous; Table S1: *E. coli*-related excreted metabolites; Table S2: Endogenous metabolites of significantly dysregulated intracellular metabolites after 24 h. of treatment with *E. coli* secretome; Table S3: Dysregulated extracellular endogenous metabolites that were secreted in culture media after treating MCF-7 cells with *E. coli* secretome; Table S4: Common metabolites between intra- and extracellular of MCF-7 cells after treatment with *E. coli* secretome.
