# Supplementary material for: Metabolic Alteration of MCF-7 Cells upon Indirect Exposure to E. coli Secretome: A Model of Studying the Microbiota Effect on Human Breast Tissue
Source: Metabolites. 2023 Aug 11;13(8):938. doi: 10.3390/metabo13080938 (PMC10456566; doi:10.3390/metabo13080938)
Supplement: Supplementary file 1 [file metabolites-13-00938-s001.zip › metabolites-2514058_Supp..pdf]

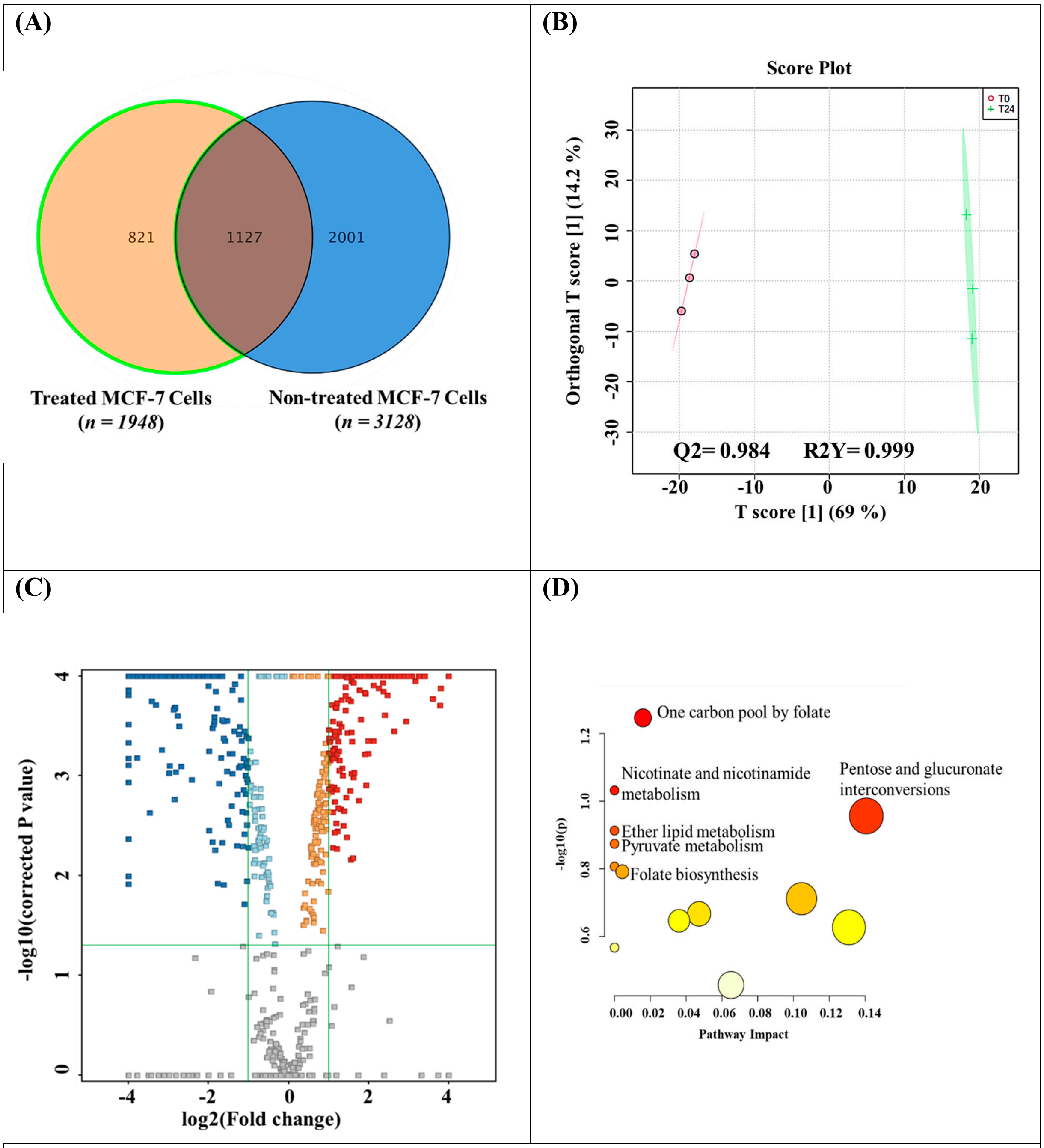

Figure S1: Dysregulated extracellular metabolites between MCF-7 cells pre-and 24hr post-treatment with *E. coli* secretome. A) A Venn diagram represents the relation between significantly dysregulated extracellular ions in treated MCF-7 with *E. coli* secretome (n=1948) and non-treated cells (n=3128) at different time points (0, 1, 2, 6, 8, and 24 hr), B) An OPLS-DA model represents the separation between pre- and 24hr post-treatment samples based on selected 821 extracellular ions. The robustness of the created model was evaluated by the fitness of the model (R2Y= 0.999) and predictive ability (Q2= 0.984) values in a larger dataset (n= 1000). C) Volcano plot revealed 437 significantly dysregulated metabolites, where 159 (in red)

and 278 (in blue) ions were up- and down-regulated in 24hr post-treatment compared to control, respectively (Cut-off: FDR $\leq$ 0.05, and FC 2), D) Pathway analysis for the significant metabolites dysregulated that secreted in culture media after treating MCF-7 cells with *E. coli* secretome. 56 metabolites were ultimately identified as endogenous.
